# Prevalence and Type Distribution of Malaria Among Children in Ethiopia: A Systematic Review and Meta‐Analysis

**DOI:** 10.1155/japr/7895997

**Published:** 2026-10-06

**Authors:** Alemayehu Abate, Solomon Alebele, Miseganaw Sisay, Tesfalem Asnake, Abaineh Munshea

**Affiliations:** ^1^ Health Research Development Directorate Amhara Public Health Institute Bahir Dar Ethiopia ephi.gov.et; ^2^ Department of Biology College of Natural and Computational Science Debre Tabor University Debre Tabor Ethiopia dtu.edu.et; ^3^ Department of Biology College of Natural and Computational Science Salale University Fiche City Ethiopia slu.edu.et; ^4^ Department of Biology Science College Bahir Dar University Bahir Dar City Ethiopia bdu.edu.et; ^5^ Health Biotechnology Division Institute of Biotechnology Bahir Dar University Bahir Dar Ethiopia bdu.edu.et

**Keywords:** children, Ethiopia, malaria, meta-analysis, *Plasmodium falciparum*, *Plasmodium vivax*, prevalence, regional variation

## Abstract

**Background:**

Malaria remains a major cause of morbidity and mortality among children in sub‐Saharan Africa. This systematic review and meta‐analysis estimated the prevalence and type distribution of malaria among children in Ethiopia and species‐specific patterns.

**Methods:**

All appropriate databases and gray literature were searched to find relevant articles. Studies reporting the prevalence of malaria among children were included. The quality of each study was assessed using the Newcastle–Ottawa Quality Assessment Scale (NOS). Data were extracted using Microsoft Excel 2016, and analysis was done using STATA 16.0 statistical software. The pooled prevalence and its associated factors of malaria were determined using a random effect model. Heterogeneity between studies was assessed using the Cochrane *Q*‐test statistics and *I* ^2^ test. Furthermore, publication bias was checked by the visual inspection of the funnel plot and using Egger′s and Begg′s statistical tests. Subgroup analyses were conducted by region and parasite species.

**Results:**

The pooled prevalence of malaria among children in Ethiopia was 17.96% (95% CI: 11.92–24.01). Subgroup analysis showed significant regional variation (*p* < 0.001), with the highest prevalence in Afar (41.90%) and the lowest in Benishangul‐Gumuz (3.90%). *Plasmodium falciparum* was the dominant species (59%), followed by *Plasmodium vivax* (35%) and mixed infections (6%). Evidence of publication bias and small‐study effects was observed; however, sensitivity analysis indicated that the overall estimate was robust. Species‐wise, pooled proportions were *P*. *falciparum* (0.59; 95% CI: 0.59–0.60), *P*. *vivax* (0.35; 95% CI: 0.34–0.35), and mixed infections (0.06; 95% CI: 0.04–0.08).

**Conclusion:**

Malaria remains a major public health challenge among Ethiopian children, with significant regional and species variation. Strengthened, region‐specific malaria control strategies are essential to reduce transmission and improve child health outcomes.

## 1. Introduction

Malaria is still the primary cause of morbidity and mortality among children in sub‐Saharan Africa [1]. According to the World Health Organization (WHO), 263 million malaria cases (95% CI: 238–294 million) occurred globally in 2023, equating to an incidence of 604 cases per 1000 population at risk, increasing from 586 cases per 1000 population at risk in 2022. A total of 94% of all global cases in 2023 occurred in Africa, with 52% of the global burden shared between just five African countries—Nigeria, Democratic Republic of the Congo, Uganda, Ethiopia, and Mozambique [2].

Ethiopia, characterized by diverse ecological and climatic conditions, remains one of the malaria‐prone countries in Africa, with seasonal and spatial variability in transmission intensity [3]. A total of 52% of the population is at risk of contracting malaria because over 75% of the country′s land is susceptible to the disease′s spread [4]. Malaria is brought on by infection with single‐celled parasites belonging to the *Plasmodium* genus. Five *Plasmodium* species are known to cause illness in humans *Plasmodium falciparum*, *Plasmodium vivax*, *Plasmodium malariae*, *Plasmodium ovale*, and *Plasmodium knowlesi*. The majority of cases of malaria worldwide are caused by *P*. *falciparum* and *P*. *vivax* [5].

Malaria remains a serious public health concern in Ethiopia among children under five. This age group′s low acquired immunity puts them at the biggest risk of infection, serious illness, and death [6]. Critical risk factors for malaria infection in children under five, including sleeping outside, not using insecticide‐treated mosquito nets (ITNs), being close to standing water, having subpar housing, and having little caregiver awareness [7].

Ethiopia has a very diverse malaria epidemiology: The severity of transmission varies by season and environment, and estimates of prevalence vary significantly between facility studies and community‐based surveys, as well as by diagnostic technique (PCR [polymerase chain reaction], RDT (rapid diagnostic test), and microscopy) [8]. Despite inconsistency among existing studies, several factors were associated with high prevalence of malaria among children under 5 years. ITN utilization, presence of forest cover, altitude of residence, household density, living near mosquito breeding sites such as stagnant water, seasonal variation, and housing conditions were major predictors of malaria infection among children aged under 5 years [9].

Significant variations in prevalence among children in particular Ethiopian settings have been reported by a number of recent primary investigations, highlighting the necessity for a comprehensive synthesis to generate more accurate, nationally applicable estimates and investigate sources of variability. The objective of this study was to determine prevalence and type distribution of malaria among children in Ethiopia. This systematic review and meta‐analysis will provide a more conclusive result to take evidence‐based interventional measures against malaria in under‐fives in Ethiopia.

## 2. Methods

### 2.1. Study Design, Period and Reporting

This systematic review and meta‐analysis was conducted to estimate the prevalence and type distribution of malaria among children in Ethiopia. Data collection, extraction, and analysis were conducted between November 2025 and February 2026. The study followed the Preferred Reporting Items for Systematic Reviews and Meta‐Analyses (PRISMA 2020) guidelines [10] and adhered to recommendations from the Cochrane Handbook for Systematic Reviews of Interventions [11].

### 2.2. Search Strategy

A thorough and methodical search of published and unpublished works up until October 2025 was done in order to find studies that reported the prevalence of malaria in Ethiopian children under five. PubMed/MEDLINE, Embase, Scopus, Web of Science, HINARI, Science Direct literature, and African Journals Online (AJOL) were among the electronic databases that were searched. Furthermore, reports from the Ethiopian Public Health Institute (EPHI) and the Federal Ministry of Health (FMOH) as well as Ethiopian institutional repositories such as Addis Ababa University, Bahir Dar University, Jimma University, and Gondar University were used to investigate gray literature. A key word arrangement that conformed to the Medical Subject Heading (MeSH) was used to choose the parameters for the search in order to find relevant papers. Boolean operators like “AND” and “OR” were used both alone and in conjunction with the search terms. An example of a search strategy used to locate relevant articles is provided here: from PubMed: ((“malaria”[MeSH terms] OR malaria OR “*Plasmodium falciparum*” OR “*Plasmodium vivax*” OR *Plasmodium malariae* OR *Plasmodium ovale*) AND (“child”[MeSH Terms] OR children OR infant OR toddler OR “under five” OR “adult children” OR preschool children) AND “Ethiopia”[MeSH terms] OR Ethiopia OR Ethiopian). From Embase: (“malaria”/exp OR malaria OR “*Plasmodium falciparum*” OR “*Plasmodium vivax*” OR *Plasmodium malariae* OR *Plasmodium ovale*) AND (“child”/exp OR child OR infant OR “under five” OR “preschool”) AND (“Ethiopia”/exp OR Ethiopia OR Ethiopian). From Web of Science: (malaria OR “*Plasmodium falciparum*” OR “*Plasmodium vivax*” OR “*Plasmodium malariae*” OR “*Plasmodium ovale*”) AND (child∗ OR infant∗ OR “under five” OR “under‐five” OR preschool∗) AND (Ethiopia OR Ethiopian). In addition to peer‐reviewed databases, we conducted a comprehensive search of unpublished and gray literature, including institutional repositories, university digital libraries, conference proceedings, and other nonpeer‐reviewed evidence sources. All retrieved articles were exported to EndNote 20 for deduplication prior to screening and eligibility assessment.

### 2.3. Eligibility Criteria

#### 2.3.1. Inclusion Criteria

Studies involving children living in Ethiopia were deemed eligible for this review. We included cross‐sectional, cohort, or surveillance studies that used validated diagnostic techniques like PCR, RDT, or microscopy to report the prevalence of malaria. To guarantee comprehensiveness, both peer‐reviewed published literature and pertinent gray literature were incorporated.

#### 2.3.2. Exclusion Criteria

Studies that were reviews, editorials, case reports, or case series were not included. To preserve the accuracy and integrity of the study, articles that represented duplicate publications or overlapping datasets, lacked defined diagnostic procedures, or contained incomplete data were also excluded.

### 2.4. Study Screening and Selection

The full texts, abstracts, and titles of the retrieved publications were examined independently by two reviewers. A third reviewer or a debate and consensus were used to settle disagreements. The selection procedure, including the number of studies found, reviewed, included, and removed with justifications, was documented using the PRISMA 2020 flow diagram.

### 2.5. Data Extraction

Data were extracted using Microsoft Excel spreadsheet imported into STATA version 16.0 statistical software for further analysis. Three authors (A.A., S.A., and M.S.) extracted the data independently. Discrepancies between authors during data extraction were managed through consensus and a fourth and fifth author (T.A. and A.M.) were consulted to resolve disagreements. For each included article, the first author′s last name, year of publication, study setting, study design, study period, sample size, and effect estimate were recorded.

### 2.6. Quality Assessment

Critical appraisal, which was integrated into the review process, was used to evaluate the quality and potential for bias prior to inclusion in the review. The Joanna Briggs Institute (JBI) Critical Appraisal Checklist for prevalence studies was used to assess the methodological quality of the included research [12]. Nine questions on the checklist evaluate representativeness, measurement reliability, sampling technique, and suitability for data processing. *Low risk* (≥ 7 points), *moderate risk* (4–6 points), and *high risk* (≤ 3 points) were assigned to each study. Reviewers′ disagreements were settled by consensus.

### 2.7. Data Synthesis and Statistical Analysis

The extracted data were analyzed using STATA Version 17.0. The pooled prevalence of malaria among children was estimated using a random‐effects model (DerSimonian and Laird method) to account for anticipated heterogeneity between studies [13]. Forest plots were created to display the prevalence of malaria reported in each study along with their 95% confidence intervals (CI) and the pooled estimate. The inverse variance method was used to determine study weights in order to account for sample size and precision variations. Using the *I* ^2^ statistic and Cochran′s *Q* statistic, between‐study heterogeneity was evaluated; *I* ^2^ values of 25%, 50%, and 75% were interpreted as low, moderate, and high heterogeneity, respectively [14]. Subgroup analyses were performed according to geography in order to investigate possible sources of heterogeneity. Egger′s regression test was used to quantitatively examine publication bias, and funnel plot symmetry was used to evaluate bias [15]. Sensitivity analyses were conducted to investigate the impact of individual studies on the overall pooled estimate by systematically eliminating one study at a time.

## 3. Results

### 3.1. Study Selection

The initial search yielded 24856 records from electronic databases and 36 additional records from gray literature. After removal of duplicates, 9362 articles remained for title and abstract screening; 9044 records were excluded after screening by title and abstracts. Of these, 318 full‐text articles were assessed for eligibility, and 72 studies met the inclusion criteria. Finally, all 18 studies were included in the final systematic review and meta‐analysis; 54 studies were excluded due to lack of outcome reports. A PRISMA 2020 flow diagram (Figure 1) summarizes the study selection process, showing the number of studies identified, screened, excluded, and included in the final analysis.

**Figure 1:**
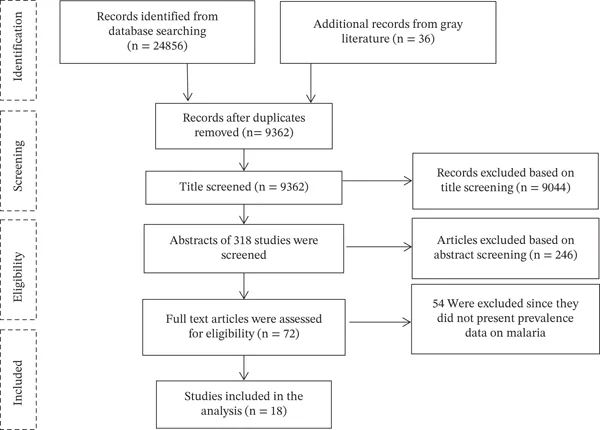
The PRISMA flow diagram of literature search and screening of included articles.

### 3.2. Characteristics of Included Studies

A total of 18 studies [16–33] were included in this systematic review and meta‐analysis assessing malaria prevalence among children in Ethiopia. The included studies comprised approximately 9916 under‐five children. All included studies employed a cross‐sectional study design and were conducted between 2017 and 2025 across different regions of Ethiopia, including Afar, Amhara, Oromia, Southern Nations, Nationalities, and Peoples′ Region (SNNPR), and Benishangul. The studies represented diverse geographical settings, with the highest number of studies originating from the Amhara region (*n* = 6), followed by SNNPR (*n* = 5), Oromia (*n* = 4), Afar (*n* = 2), and Benishangul (*n* = 1). Sample sizes varied considerably across studies, ranging from 271 participants in studies by Legesse et al. [29] and Abossie et al. [22] to 3075 participants in Elias et al. [24]. The characteristics and individual results of these studies are summarized in Table 1.

**Table 1:** Characteristics and individual results of studies included in the meta‐analysis on the prevalence of malaria among children in Ethiopia.

Author	Study region	Study design	Sample size	Positive case	Prevalence
File and Jemal [16]	Afar	Cross‐sectional	389	77	19.8
Duguma et al. [17]	SNNPR	Cross‐sectional	286	67	23.4
Cherkos et al. [19]	Amhara	Cross‐sectional	357	33	9.2
Aliyo et al. [20]	Oromia	Cross‐sectional	437	117	27.8
Elias et al. [24]	SNNPR	Cross‐sectional	3,075	59	1.9
Alemu et al. [25]	Oromia	Cross‐sectional	466	57	12.23
Lemma et al. [23]	Oromia	Cross‐sectional	557	28	5.2
Tsegaye et al. [18]	Amhara	Cross‐sectional	585	51	8.7
Debash et al. [21]	Amhara	Cross‐sectional	633	156	24.6
Worinesh et al. [28]	Amhara	Cross‐sectional	422	62	14.7
Worinesh et al. [28]	Amhara	Cross‐sectional	207	18	8.7
Merkeb et al. [34]	Oromia	Cross‐sectional	281	7	2.5
Dagnew et al. [26]	Amhara	Cross‐sectional	420	173	41.2
Woday et al. [35]	Afar	Cross‐sectional	484	310	64.04
Legesse et al. [29]	SNNPR	Cross‐sectional	271	66	24.32
Ahmed et al. [27]	Benishangul	Cross‐sectional	356	14	3.93
Abossie et al. [22]	SNNPR	Cross‐sectional	271	60	22.14
Abrham et al. [36]	SNNPR	Cross‐ sectional	419	38	9.1

### 3.3. Pooled Prevalence of Malaria Among Children

A total of 18 studies were included in the meta‐analysis. The individual effect sizes ranged from 1.90% (95% CI: 1.85–1.95) in Elias et al. [24] to 64.00% (95% CI: 63.96–64.04) in Woday et al. [35]. The pooled overall effect using a random‐effects model (DerSimonian–Laird method) was 17.96% (95% CI: 11.92–24.01). There was substantial heterogeneity among the studies (*I* ^2^ = 100.0*%*, *p* < 0.001) (Figure 2).

**Figure 2:**
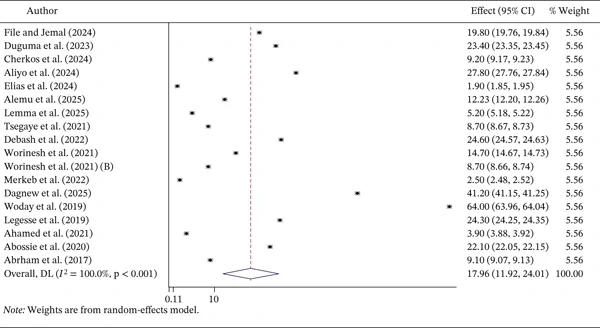
Forest plot analysis: The pooled estimates of the prevalence of malaria among children in Ethiopia.

### 3.4. Publication Bias and Sensitivity Analysis

Visual inspection of the funnel plot demonstrated a slight asymmetry in the distribution of studies around the pooled effect estimate. The funnel plot suggested possible evidence of publication bias; however, visual interpretation alone is insufficient for definitive conclusions. Therefore, additional statistical tests such as Egger′s regression test and Begg′s rank correlation test were performed to further assess publication bias. Publication bias among the included studies was assessed using Egger′s regression asymmetry test. The findings indicated statistically significant evidence of small‐study effects and potential publication bias among the studies included in the meta‐analysis. The Egger′s regression intercept (bias coefficient) was 1654.61 (SE = 445.03, *t* = 3.72, *p* = 0.002; 95% CI: 711.18–2598.03), demonstrating significant funnel plot asymmetry. The slope coefficient was −12.44 (SE = 7.01, *t* = −1.77, *p* = 0.095; 95% CI: −27.30 to 2.43), which was not statistically significant. However, the overall test for small‐study effects was statistically significant (*p* = 0.002), leading to rejection of the null hypothesis of no publication bias (Figure 3 and Table 2).

**Figure 3:**
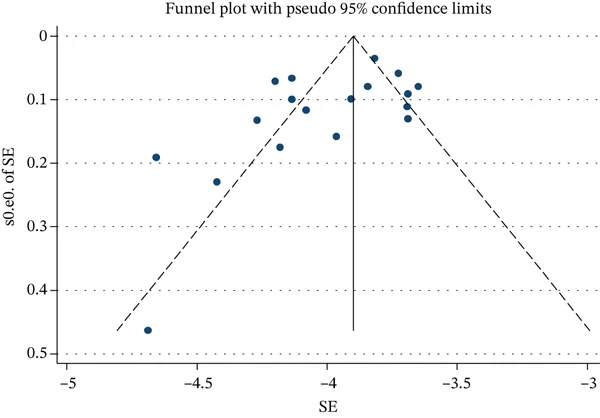
Funnel plot of the prevalence of malaria among children in Ethiopia.

**Table 2:** Egger′s test of funnel plot asymmetry and small‐study effects.

Std_Eff	Coefficient	Standard error	*T*	*p* > *t*	95% confidenceinterval	
Slope	−12.43612	7.01319	−1.77	0.095	−27.30342	2.43118
Bias	1654.606	445.0308	3.72	0.002	711.1829	2598.029

*Note:* Test of H0: No small‐study effects, *p* = 0.002.

### 3.5. Sensitivity Analysis

A leave‐one‐out sensitivity analysis was conducted to assess the influence of individual studies on the pooled estimate. The results showed that omitting each study one at a time did not substantially alter the overall effect size. The pooled estimates ranged from 15.25 to 18.91, whereas the corresponding 95% CIs remained largely overlapping with the overall pooled estimate. The lowest pooled estimate was observed when Woday et al. [35] was excluded (15.25; 95% CI: 10.80–19.71), whereas the highest estimate occurred when Elias et al. [24] was omitted (18.91; 95% CI: 12.66–25.16). Despite these variations, the overall pooled effect size remained stable at 17.96 (95% CI: 11.92–24.01) (Table 3).

**Table 3:** Sensitivity analysis showing the influence of individual studies on the pooled prevalence of malaria among children in Ethiopia.

Study omitted	Pooled estimate	95% confidence interval
File and Jemal [16]	17.85	11.55–24.16
Duguma et al. [17]	17.64	11.40–23.89
Cherkos et al. [19]	18.48	12.05–24.90
Aliyo et al. [20]	17.38	11.21–23.56
Elias et al. [24]	18.91	12.66–25.16
Alemu et al. [25]	18.3	11.85–24.75
Lemma et al. [23]	18.71	12.15–25.28
Tsegaye et al. [18]	18.51	12.10–24.91
Debash et al. [21]	17.57	11.34–23.81
Worinesh et al. [28]	18.15	11.76–24.55
Worinesh et al. [28]	18.51	12.16–24.86
Merkeb et al. [34]	18.87	12.49–25.26
Dagnew et al. [26]	16.6	10.72–22.48
Woday et al. [35]	15.25	10.80–19.71
Legesse et al. [29]	17.59	11.36–23.82
Ahmed et al. [27]	18.79	12.38–25.20
Abossie et al. [22]	17.72	11.46–23.98
Abrham et al. [36]	18.48	12.02–24.95
Combined	17.96	11.92–24.01

### 3.6. Subgroup Analysis by Region

A subgroup analysis was conducted to explore regional variations in the prevalence of malaria among under‐five children in Ethiopia. The findings revealed substantial variation in malaria prevalence across different regions, with statistically significant heterogeneity observed between subgroups (*p* < 0.001). The highest pooled prevalence was observed in the Afar region, with an estimated prevalence of 41.90% (95% CI: −1.42% to 85.22%), based on two studies. Studies from the SNNPR reported a pooled prevalence of 16.16% (95% CI: 7.74%–24.58%). In the Amhara region, the pooled prevalence was 17.85% (95% CI: 9.18%–26.52%), whereas studies conducted in Oromia demonstrated a lower pooled prevalence of 11.93% (95% CI: 3.87%–20.00%). The lowest prevalence estimate was reported from Benishangul, with a prevalence of 3.90% (95% CI: 3.88%–3.92%) (Figures 4 and 5).

**Figure 4:**
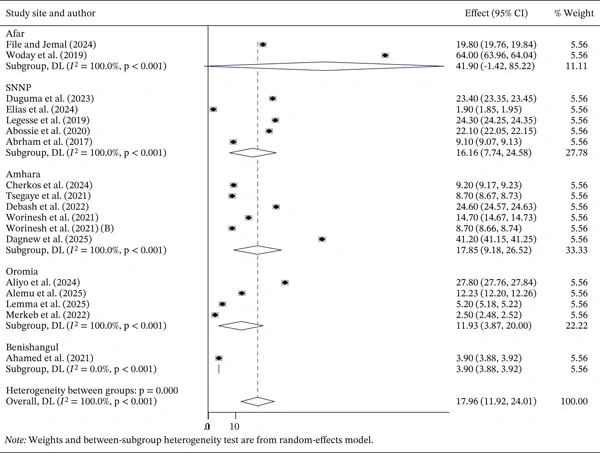
Subgroup analysis of malaria prevalence among children in Ethiopia stratified by region.

**Figure 5:**
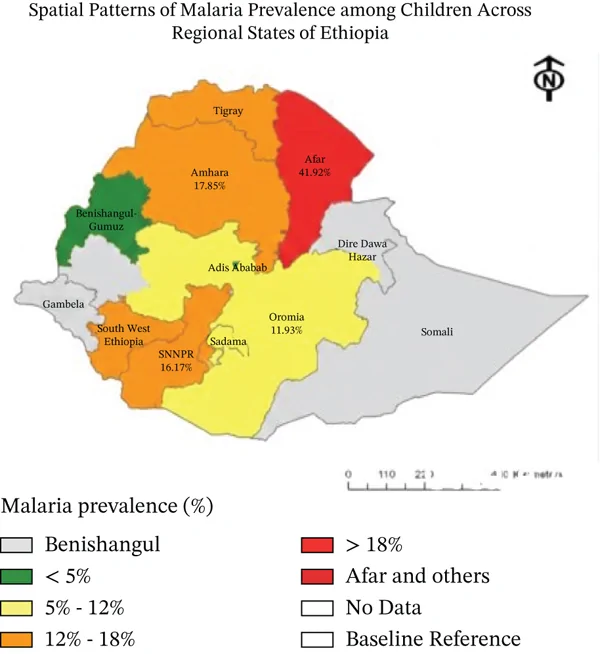
Spatial distribution of malaria among children across regional states of Ethiopia.

### 3.7. Proportion of *P*. *falciparum*

A total of 15 studies were included in this meta‐analysis to estimate the pooled proportion of *P*. *falciparum*. The overall pooled prevalence was 0.59 (95% CI: 0.59–0.60). The individual study proportion estimates ranged from 0.45 to 0.86, reflecting considerable differences across study settings. Despite this, the combined result suggests that malaria remains prevalent in the studied populations (Figure 6).

**Figure 6:**
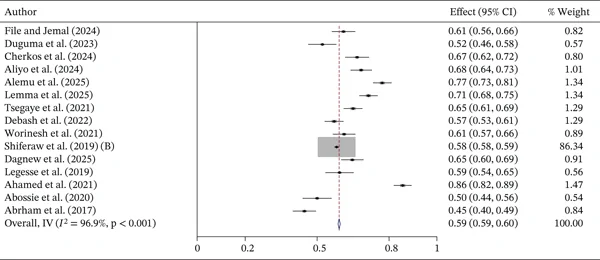
Proportion of *P*. *falciparum* among children in Ethiopia.

### 3.8. Proportion of *P*. *vivax*

A total of 15 studies were included in the pooled analysis. The overall pooled proportion of *P*. *vivax* was 0.35 (95% CI: 0.34–0.35) using the inverse‐variance random‐effects model. The prevalence estimates across the included studies ranged from 0.14 to 0.48, indicating notable variation in malaria burden across study populations and regions (Figure 7).

**Figure 7:**
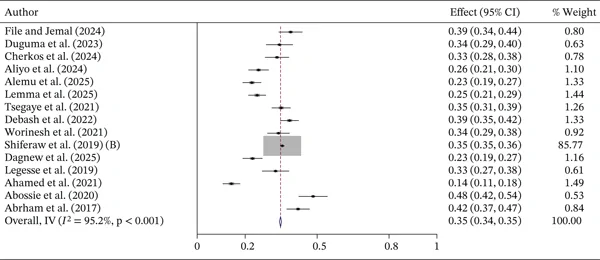
Proportion of *P*. *vivax* among children in Ethiopia.

### 3.9. Proportion of Mixed Infection

A total of 10 studies were included in the pooled meta‐analysis. The overall pooled proportion of Mixed (Both *P*. *falciparum* and *P*. *vivax)* malaria was 0.06 (95% CI: 0.04–0.08). Individual study estimates ranged from approximately 0.04 to 0.13 (Figure 8).

**Figure 8:**
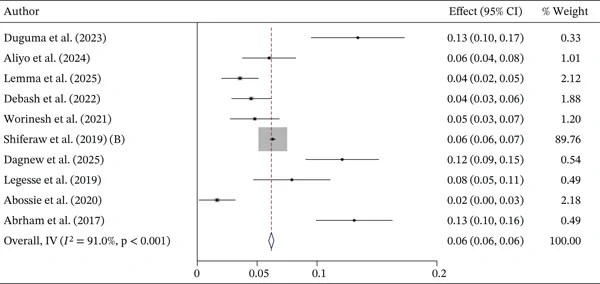
Proportion of mixed infections among children in Ethiopia.

### 3.10. Subgroup Analysis by Year

The pooled prevalence of malaria among included studies was 21.0% (95% CI: 14.16–27.85). However, substantial heterogeneity was observed among studies (*I* ^2^ = 100*%*, *p* < 0.001), indicating considerable variability in prevalence estimates across settings. Subgroup analysis by publication year showed marked differences in prevalence, ranging from 5.2% to 64.0%. The reported prevalence showed considerable variation across studies, ranging from approximately 5%–9% in studies conducted in 2017, 2021, and 2025 to as high as 64% in the study by Woday et al. [35]. However, the majority of studies published after 2020 reported prevalence estimates within the range of 10%–25% (Figure 9).

**Figure 9:**
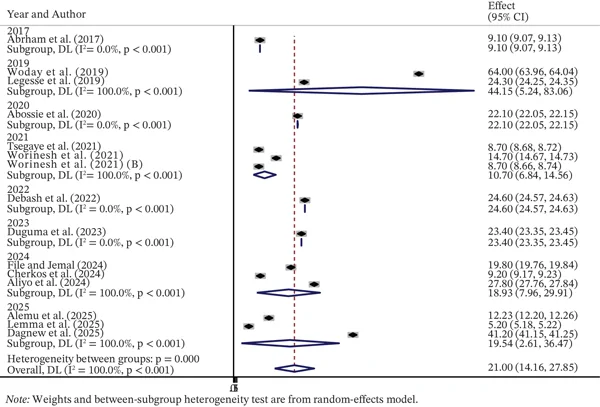
Prevalence of malaria among children in Ethiopia across years.

## 4. Discussion

This systematic review and meta‐analysis synthesized evidence on the prevalence and type distribution of malaria among children in Ethiopia. A total of 18 studies, with 47,642 participants from regions, were included in this analysis. Malaria infection is influenced by geography, season, age, socioeconomic status, environmental conditions, and changing transmission patterns [31]. Globally, under‐five children account for a substantial proportion of malaria‐related morbidity and mortality. In high‐transmission settings [32]. malaria is a leading cause of outpatient visits, hospital admissions, and preventable childhood deaths [33]. Their infection risk depends on transmission intensity, acquired immunity, intervention coverage, and age [37]. Despite often being asymptomatic, school‐aged children remain a key reservoir sustaining malaria transmission [38].

In this systematic review and meta‐analysis, the pooled prevalence of malaria among children was 17.96% (95% CI: 11.92–24.01) using the random‐effects model (DerSimonian and Laird method). This result was consistent with several regional findings: A systematic review of asymptomatic Malaria Infection in Children in Sub‐Saharan Africa reported that the pooled prevalence of under‐five malaria was 22.03% (95% CI: 12.25%, 31.80%) [39]; a study reported that the pooled prevalence of under‐five malaria was 26.2% [40]; a multilevel analysis using recent malaria indicator surveys of sub‐Saharan African countries reported that the pooled prevalence of malaria among children aged 6–59 months was found to be 27.41% (95% CI: 17.94%–36.88%) [41]; and a study from Kenya reported that the prevalence of malaria among under‐five children in Kenya was 22.72% (95% CI: 21.29, 24.22) [42]. This consistency across age groups and countries in East Africa indicates a regional challenge of sustained malaria transmission that affected children. However, the observed pooled prevalence is relatively lower than other parts of the continent can be significantly higher. A 2023 geospatial analysis in eight West African countries, for example, identified Benin and Côte d′Ivoire as high‐burden areas with predicted prevalence frequently exceeding 30% [43].

The subgroup analysis demonstrated marked regional variation in the prevalence of malaria among under‐five children in Ethiopia, with statistically significant heterogeneity across regions (*p* < 0.001). The highest pooled prevalence was observed in the Afar region (41.90%), followed by Amhara (17.85%) and SNNPR (16.16%), whereas Oromia showed a comparatively lower prevalence (11.93%) and Benishangul‐Gumuz the lowest (3.90%). These results are consistent with Ethiopian national surveillance that reported malaria cases in Amhara were 18% with 56 deaths, Southwest Ethiopia (12% of cases, 250 deaths), and the SNNPR (7% of cases, 45 deaths). The lowland terrain and climatic conditions of Afar and Amhara, which provide perfect circumstances for *Anopheles* mosquito growth and malaria transmission, were major contributors to the high transmission in these regions [44]. In order to facilitate persistent transmission, *Anopheles arabiensis* and *Anopheles stephensi* benefit from ideal breeding conditions created by ecological factors such semiarid regions, seasonal rainfall, and proximity to irrigation schemes [45]. The substantial heterogeneity observed across the regions is likely attributable to differences in malaria transmission intensity across Ethiopia. Malaria transmission in the country is highly heterogeneous, varying by altitude, climate, rainfall patterns, ecological conditions, seasonality, and the distribution of mosquito vectors. Regions with stable or higher transmission generally report greater malaria prevalence than low‐transmission or epidemic‐prone highland areas. In addition, differences in study periods may reflect changes in malaria epidemiology resulting from the scale‐up of vector control interventions, improvements in case management, and fluctuations in environmental conditions. Consequently, the pooled prevalence should be interpreted as an overall estimate that encompasses diverse epidemiological settings rather than representing a uniform malaria burden across Ethiopia.

Visual examination of the funnel plot revealed asymmetry, which may indicate publication bias or small‐study effects. The statistically significant Egger′s regression test (intercept = −8.07, *p* = 0.024) showed small‐study effects, and the Begg′s test (*p* = 0.002) also raised the possibility of bias. Both tests have low specificity when there is extreme heterogeneity, so these results should be interpreted with caution [46]. The results are robust overall, as demonstrated by sensitivity analysis employing the “leave‐one‐out” method, which revealed that eliminating individual studies had no discernible impact on the pooled estimate.

This meta‐analysis estimated the pooled prevalence of *P*. *falciparum*, *P*. *vivax*, and mixed infections in Ethiopian children*. P*. *falciparum*′s overall pooled prevalence was 59% (95% CI: 59–60). This finding was in line with an Ethiopian study that found *P*. *falciparum* to be the most common parasite in the villages, primarily affecting children [24]. For *P*. *vivax*, the pooled prevalence was 35% (95% CI: 34–35), whereas mixed infections accounted for 6% (95% CI: 4–8). These findings were similar with a systematic review and meta‐analysis *of P*. *vivax* epidemiology in Ethiopia that reported among screened 4932 records and included 79 studies that enrolled 1,676,659 confirmed malaria cases, from which 548,214 (32.69%) were *P*. *vivax* infections and 1,116,581 (66.59%) were due to *P*. *falciparum*. The rest were due to mixed infections [47]. Despite being less frequent, the presence of mixed infections (6%), presented difficulties for control and treatment plans. Because *P*. *vivax* and *P*. *falciparum* have different biological traits and treatment plans, mixed infections can make diagnosis more difficult and may call for different therapeutic approaches. This outcome aligns with a meta‐analysis and systematic review. 9% (2,006/35,768, 95% CI: 7.0%–12.0%) of patients with mixed *Plasmodium* infection had severe mixed infection, according to a pooled analysis of *Plasmodium* spp. mixed infection resulting in severe malaria [48].

The observed discrepancy in prevalence estimates between studies could be explained by variations in research environments, diagnostic techniques, and demographics. Studies in highland areas may reveal lower prevalence due to decreased vector activity, whereas favorable conditions for mosquito breeding may lead to higher rates in lowland areas [49–54]. These findings highlight the importance of putting in place all‐encompassing, situation‐specific policies that cover nutritional interventions, vector control, chemoprophylaxis, and health system enhancement. To safeguard children′s health and lower the prevalence of malaria among children in Ethiopia and other endemic regions, it is imperative to expand intermittent preventive treatment for children (IPTc) coverage, enhance the use of insecticide‐treated nets, strengthen active case detection, and promote community engagement.

## 5. Limitations of the Study

This systematic review and meta‐analysis has some limitations that should be considered when interpreting the findings. First, substantial between‐study heterogeneity was observed, which may be explained by differences in malaria transmission intensity, ecological and climatic conditions, study populations, diagnostic methods, and study periods across the included studies. Although subgroup and sensitivity analyses were performed to explore sources of heterogeneity, some variability remained unexplained. Second, the number of studies included from certain regions, particularly Afar and Benishangul‐Gumuz, was limited, which may reduce the precision and generalizability of regional pooled estimates. This was reflected in wide CIs for some subgroup analyses. Third, the presence of publication bias and small‐study effects, as indicated by funnel plot asymmetry and Egger′s and Begg′s tests, suggests that smaller or nonsignificant studies may be underrepresented in the literature. Finally, variation in diagnostic techniques (microscopy, RDTs, or mixed methods) and differences in case definitions across studies may have introduced measurement bias, potentially affecting comparability of results. Despite these limitations, the findings provide valuable pooled evidence on the burden and distribution of malaria among under‐five children in Ethiopia.

## 6. Conclusion

The overall pooled prevalence was 17.96% (95% CI: 11.92–24.01). *P*. *falciparum* was responsible for the majority of infections (59%), followed by *P*. *vivax* (35%) and mixed infections (6%). Regional disparities were evident as the highest prevalence was found in the Amhara and Afar regions. The persistence of malaria in children highlights the need for context‐specific, integrated interventions that combine chemoprophylaxis, vector control, and strengthening of the health system. Increased use of insecticide‐treated nets, expanded coverage of intermittent IPTc, enhanced active case detection, and community engagement are all necessary to stop the spread of malaria and safeguard the health of children in Ethiopia and other endemic areas.

**Nomenclature**
EPHI: Ethiopian Public Health Institute
FMOH: Federal Ministry of Health
IPT: intermittent preventive treatment
IRS: indoor residual spraying
ITNs: insecticide‐treated nets
JBI: Joanna Briggs Institute
MeSH: Medical Subject Headings
PCR: polymerase chain reaction
PRISMA: Preferred Reporting Items for Systematic Reviews and Meta‐Analyses
RDTs: rapid diagnostic tests
WHO: World Health Organization

## Author Contributions

A.A., A.M., S.A., M.S., and T.A. conceived the study and developed the research protocol. A.A., A.M., and S.A. conducted the literature search and data extraction. M.S. and T.A. performed the methodological quality assessment and statistical analysis. All authors contributed to the interpretation of the findings and preparation and revision of the manuscript.

## Funding

No funding was received for this research.

## Disclosure

All authors read and approved the final version of the manuscript.

## Ethics Statement

The authors have nothing to report.

## Consent

The authors have nothing to report.

## Conflicts of Interest

The authors declare no conflicts of interest.

## Data Availability

All materials and data related to this article are included in the main document of the manuscript.
